# Involvement of Thyroid Hormones in the Expression of MHC class I  Antigens During Ontogeny in *Xenopus*

**DOI:** 10.1155/1997/38464

**Published:** 1997

**Authors:** Louise A. Rollins-Smith, Martin F. Flajnik, Patrick J. Blair, A. Tray Davis, Wayne F. Green

**Affiliations:** 1 Departments of Microbiology & Immunology, Pediatrics Vanderbilt University School of Medicine Nashville Tennessee 37232 USA; 2 Department of Pathology Vanderbilt University School of Medicine Nashville Tennessee 37232 USA; 3 Department of Microbiology and Immunology University of Miami School of Medicine Miami Florida 33101 USA

**Keywords:** MHC class I, Metamorphosis, Thyroid Hormones, Xenopus

## Abstract

The major histocompatibility complex (MHC) is a cluster of genes encoding products central to all major functions of the vertebrate immune system. Evidence for an MHC can be found in all vertebrate groups that have been examined except the jawless fishes. Expression of MHC class I and class II antigens early in ontogeny is critically important for development of T lymphocytes capable of discriminating self from nonself. Because of this essential role in T-cell development, the ontogeny of MHC expression in the South African clawed frog, *Xenopus laevis*, was studied. Previous studies of MHC class I expression in *Xenopus laevis* suggested that class I antigens are virtually absent from tadpole tissues until climax of metamorphosis. We therefore examined the possible role of thyroid hormones (TH) in the induction of class I. By flow cytometry, a small amount of class I expression was detectable on splenocytes and erythrocytes in untreated frogs at prometamorphic stages 55-58, and the amount increased significantly at the conclusion of metamorphic climax. Thus, metamorphosis is associated with increased intensity of class I expression. Neither inhibition nor acceleration of metamorphosis altered the timing of onset of class I expression. However, inhibition of metamorphosis prevented the increase in class I expression characteristic of adult cell populations. Because expression was not accelerated in TH-treated frogs or delayed in metamorphosis-inhibited frogs, it is unlikely that TH are the direct developmental cues that induce expression, although they seem to be required for the upregulation of class I expression occurring at metamorphosis. Differences in the pattern of expression in different subpopulations of cells suggest a complex pattern of regulation of expression of class I antigens during ontogeny.


*Developmental Immunology*, 1997, Vol. 5, pp. 133–144
Reprints available directly from the publisher
Photocopying permitted by license only

© 1997 OPA (Overseas Publishers Association)
Amsterdam B.V. Published under license in The Netherlands
under the Harwood Academic Publishers imprint
part of The Gordon and Breach Publishing Group
Printed in Malaysia

### Involvement of Thyroid Hormones in the Expression of MHC Class I Antigens During Ontogeny in *Xenopus*

LOUISE A. ROLLINS-SMITH*[†], MARTIN F. FLAJNIK[§], PATRICK J. BLAIR[†], A. TRAY DAVIS[†], AND WAYNE F. GREEN[‡]

[†]*Departments of Microbiology & Immunology, Pediatrics, and* [‡]*Pathology, Vanderbilt University School of Medicine, Nashville, Tennessee 37232;* [§]*Department of Microbiology and Immunology, University of Miami School of Medicine, Miami, Florida 33101*

*(Received 8 February 1996; Accepted 8 March 1996)*

The major histocompatibility complex (MHC) is a cluster of genes encoding products central to all major functions of the vertebrate immune system. Evidence for an MHC can be found in all vertebrate groups that have been examined except the jawless fishes. Expression of MHC class I and class II antigens early in ontogeny is critically important for development of T lymphocytes capable of discriminating self from nonself. Because of this essential role in T-cell development, the ontogeny of MHC expression in the South African clawed frog, *Xenopus laevis*, was studied. Previous studies of MHC class I expression in *Xenopus laevis* suggested that class I antigens are virtually absent from tadpole tissues until climax of metamorphosis. We therefore examined the possible role of thyroid hormones (TH) in the induction of class I. By flow cytometry, a small amount of class I expression was detectable on splenocytes and erythrocytes in untreated frogs at prometamorphic stages 55–58, and the amount increased significantly at the conclusion of metamorphic climax. Thus, metamorphosis is associated with increased intensity of class I expression. Neither inhibition nor acceleration of metamorphosis altered the timing of onset of class I expression. However, inhibition of metamorphosis prevented the increase in class I expression characteristic of adult cell populations. Because expression was not accelerated in TH-treated frogs or delayed in metamorphosis-inhibited frogs, it is unlikely that TH are the direct developmental cues that induce expression, although they seem to be required for the upregulation of class I expression occurring at metamorphosis. Differences in the pattern of expression in different subpopulations of cells suggest a complex pattern of regulation of expression of class I antigens during ontogeny.

*Keywords:* MHC Class I; Metamorphosis; Thyroid Hormones; *Xenopus*

### INTRODUCTION

Because amphibians evolved to exploit temporary ponds as a developmental niche for their larval stages, a functional immune system that develops quickly is necessary to enable them to respond to potential pathogens in that environment. However, studies of the ontogeny of the immune system of *Xenopus laevis* (reviewed in Flajnik et al., 1987; Du Pasquier et al., 1989; Horton, 1994; Rollins-Smith and Cohen, 1995) have demonstrated that the early immune system is not the fully developed mature immune system that will serve adult frogs. There are at least two important periods of lymphocyte expansion (Turpen and Smith, 1989; Rollins-

*Corresponding author.

Smith et al., 1992) and induction of tolerance to self. They are the early larval period, when tolerance to tadpole antigens develops, and the postmetamorphic period, when tolerance to newly developing adult-specific molecules develops. Studies in murine systems (reviewed in Schwartz, 1984) have demonstrated that the major histocompatibility complex (MHC) is intimately involved in the selection of T lymphocytes for recognition of self and recognition of foreign antigens in the context of self. In *Xenopus*, studies involving the fusion of the anterior of one embryo to the posterior of another embryo have revealed that all T-cell precursors pass through the thymus, and resulting mature T cells are "educated" to recognize self and foreign antigens in the context of the MHC expressed by the thymus (Flajnik et al., 1985). Because of the importance of the MHC in the development of immunological tolerance, it is important to understand when in ontogeny MHC antigens are expressed and what regulates their expression.

In the last ten years, the MHC of *Xenopus* has been well characterized (reviewed in Flajnik and Du Pasquier, 1990). Class I and class II molecules have been described (Flajnik et al., 1984; Kaufman et al., 1985a, 1985b; Flajnik et al., 1986) and cloned (Flajnik et al., 1991a; Sato et al., 1993; Shum et al., 1993). Monoclonal antibodies have been produced that bind specifically to each class of MHC antigens (Flajnik et al., 1990, 1991b). By using these reagents, it was possible to examine the ontogeny of expression of MHC antigens. Several distinct differences in the larval and adult patterns of expression have been described. Class II antigens are expressed predominantly on B lymphocytes and accessory cells in the tadpole, as is the pattern in most mammalian species. In addition, class II seems to have expanded tissue distribution on nonhematopoietic tissues in the tadpole (Du Pasquier and Flajnik, 1990). After metamorphosis, however, class II is expressed on virtually all mature lymphocytes including T cells (Du Pasquier and Flajnik, 1990; Rollins-Smith and Blair, 1990a). The most striking difference in MHC expression between tadpoles and adults is the apparent absence of expression of class I antigens during larval life. Expression of class I on erythrocytes begins around the time of metamorphosis (Flajnik and Du Pasquier, 1988).

Because thyroid hormones (TH) are the master hormones driving metamorphosis, it was reasonable to ask whether TH are involved in the regulation of class I expression during ontogeny. If the surge of TH associated with metamorphosis (Leloup and Buscaglia, 1977) provides the cue for class I expression, then total inhibition of TH release using goitrogens or premature treatment with exogenous TH to accelerate metamorphosis should inhibit class I expression or alter the timing of class I expression. Here we report that class I expression is first detected at low levels in the spleen at prometamorphic stages 55–56 (about 34 days of age). The level of expression is upregulated in the spleen and on erythrocytes beginning at the end of metamorphic climax (stages 65–66). Metamorphosis-inhibited tadpoles expressed low levels of class I comparable to that of normal larvae, but were unable to upregulate expression to adult levels. Thyroidectomized frogs, driven to metamorphose precociously, initially expressed low levels of class I but were eventually able to achieve adult levels of class I expression. This observation suggests that the developmentally regulated increase in class I expression is dependent on events occurring during normal metamorphosis but is not directly dependent on the elevated levels of thyroid hormones necessary to bring about metamorphic transition.

### RESULTS

### General Experimental Design

Each family of tadpoles to be examined for development of MHC class I expression was divided into three groups. One group was allowed to develop normally at 18–22°C. A second group, housed under identical experimental conditions, was treated with sodium perchlorate to inhibit metamorphosis, as described in Materials and Methods. A third group was thyroidectomized (thyroidx) and treated with increasing concentrations of thyroxine ($T_4$) to accelerate metamorphosis, as described in Materials and Methods.

At a number of developmental stages between 53–54 and 1–3 months postmetamorphosis in the untreated controls, animals from each of the three groups were sacrificed. Developmental stages were determined according to the normal table of Nieuwkoop and Faber (1967). Thymocytes, splenocytes, and erythrocytes were collected, stained, and analyzed for expression of MHC class I antigens by conventional fluorescence microscopy or by flow cytometry.

### Expression of Class I MHC Antigens Detected by Conventional Fluorescence Microscopy in Outbred Frogs

In a family of outbred frogs whose parents had been selected for expression of the class I antigen recognized by the TB17 monoclonal antibody (Flajnik et al., 1991b), there was little or no class I expression detected on splenocytes until stage 58 (day 50). About 45% of cells with the morphology of lymphocytes expressed class I at this stage (Fig. 1, upper right panel). Expression was detected on a greater proportion of splenocytes at later stages. Little or no class I expression could be detected on thymocytes until climax of metamorphosis (stages 64–65, days 61–86), and expression was increased after metamorphosis (Fig. 1, lower left panel). The pattern of developmental progression for untreated controls, $T_4$-accelerated, and perchlorate-treated animals is shown in the upper left panel of Fig. 1. In this family, the $T_4$-acceleration protocol was modified (see legend for Fig. 1) in an attempt to force metamorphosis more gradually to promote greater survival in this group. However, the result was that $T_4$-accelerated frogs advanced through metamorphosis only slightly more quickly than controls. Although the percent of class I positive splenocytes in $T_4$-accelerated frogs was slightly higher at stage 62–63 than the age-matched untreated controls, the difference reflects a single data point for the controls and the average of two determinations for the experimentals. Because *Xenopus* tadpoles develop quite rapidly, it was not possible to take repeat measurements at each stage. Perchlorate

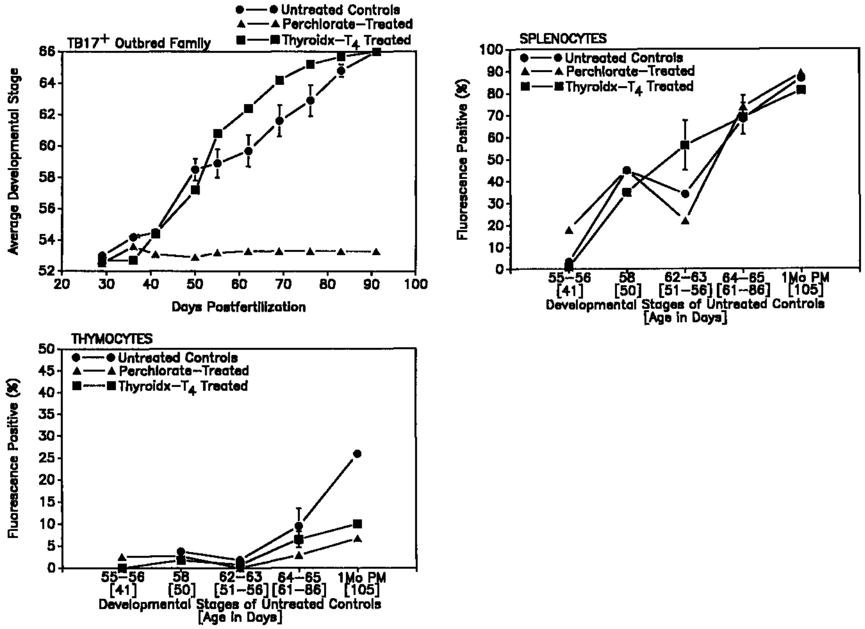

FIGURE 1 Expression of MHC class I antigens detected by conventional fluorescence microscopy in a family of outbred frogs whose parents were selected for expression of the class I antigen recognized by the TB17 monoclonal antibody. Upper left shows the developmental stage progression of normal controls (closed circles), perchlorate-blocked tadpoles (closed triangles), and thyroidx-$T_4$-accelerated frogs (closed squares). The $T_4$-treatment regimen for this group was modified slightly from the protocol described in Materials and Methods. It was as follows: Days 21–28, 0.1 $\mu$g/l $T_4$; days 28–35, 0.3 $\mu$g/l $T_4$; days 35–42, 0.5 $\mu$g/l $T_4$; days 42–49, 1 $\mu$g/l $T_4$; days 49–63, 10 $\mu$g/l $T_4$; days 63 onward, 5 $\mu$g/l $T_4$. Upper right and lower left show the percent of cells with morphology of lymphocytes showing surface fluorescence in spleen and thymus, respectively. Most data points represent a single pool of cells from 1–3 individuals except at days 61–86, which represent 4–5 pools of cells examined during that time period.

treatment prevented tadpoles from advancing beyond stage 53. Yet, the pattern of expression of MHC class I antigens on splenocytes and thymocytes of these frogs was nearly identical to that of age-matched untreated controls. Thus, inhibition of metamorphosis did not delay the onset of expression of class I antigens (Fig. 1, upper right and lower left panels).

### Expression of Class I Antigens Detected by Flow Cytometry in the MHC Homozygous F-Strain

Figure 2 shows the pattern of staining of splenocytes from stage 57 (day 86) F-strain tadpoles. The profile of viable cells represented as forward scatter (FCS) versus viability (7-AAD staining) is designated region 1 (R1) and is shown in the upper and lower left panels. The population of cells selected for fluorescence analysis is encircled (R2) in the upper and lower middle panels and generally represents small lymphocytes. In the upper right panel is shown the fluorescence pattern of splenocytes stained with an irrelevant isotype control antibody ($IgG_1$, k) and FITC-Gt-anti-mouse Ig. Nonspecific binding was 2.4% in this sample. The lower right panel shows the staining pattern with TB17 monoclonal followed by FITC-Gt-anti-mouse Ig. Approximately 62.3% of cells were fluorescence-positive. The determination of fluorescence-positive cells corrected for nonspecific staining was, therefore, 59.9%.

Figure 3 shows the developmental pattern and class I expression pattern of a family of homozygous F-strain frogs. These animals were reared in the summertime in an air-conditioned laboratory. Water temperature averaged about 18–20°C. As a result, they developed more slowly than the previous family shown in Fig. 1. $T_4$-treated frogs metamorphosed about 80 days earlier than most of the controls. Perchlorate-treated animals did not advance beyond stage 53.

At the earliest stages examined (stages 53–54, day 53), about 17% of splenocytes from untreated controls

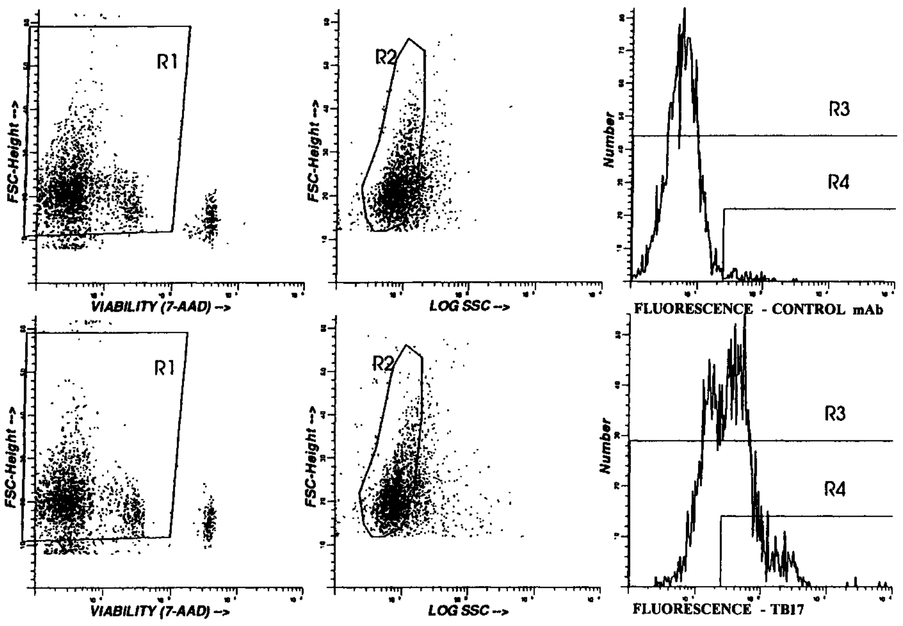

FIGURE 2 Flow cytometry profiles of spleen cells from F-strain tadpoles (stage 57, day 86). Upper panels represent cells stained with an irrelevant isotype-matched control mAb. Lower panels represent cells stained with TB17. Upper and lower left panels show the distribution of cells based on forward scatter (FSC) and viability (7-AAD Staining). Upper and lower middle panels show the distribution of cells based on FSC and log side scatter (SSC). Cells were gated on viability (region 1 = R1) as well as forward and side scatter. The population of cells examined (small lymphocytes) is designated region 2 (R2). Upper and lower right panels show fluorescence intensity on the $x$-axis and number of events per channel on the $y$-axis. Linear mean fluorescence was determined for cells in region 3 (R3), which includes all events at all channels, or region 4 (R4), which represents fluorescence positive cells.

expressed class I. That percentage increased at later stages and remained high throughout the period of study (3 months postmetamorphosis). Neither acceleration of metamorphosis nor inhibition of metamorphosis altered the time of onset or percent of cells expressing class I in the spleen. Expression on thymocytes was quite low until after metamorphosis. Perchlorate treatment prevented the increase in the percentage of positive cells observed in thymocytes from postmetamorphic controls. Few erythrocytes expressed class I until stage 57 (day 86). Increased expression continued to be observed during climax of metamorphosis, and a further increase was detected after metamorphosis. Perchlorate treatment did not alter the time of onset or the percent of erythrocytes expressing class I.

A second family of F X Outbred frogs was $T_4$-accelerated. These frogs and untreated controls were reared at 22–24°C and developed according to the Nieuwkoop and Faber schedule of stages (Nieuwkoop and Faber, 1967). The $T_4$-treated group metamorphosed about 25 days before most of the control frogs. Splenocytes, thymocytes, and erythrocytes removed from untreated animals at 27 days of age (stages 53–54) were essentially negative for class I expression. One week later at stages 55–56 (day 34), about 45% of splenocytes were class I positive. As was seen with homozygous F-strain animals, the percent of class I expressing cells increased at later time points. Acceleration of metamorphosis did not alter the pattern of expression in the spleen (data not shown). Thymocytes did not show class I expression until day 53 (stage 60) and acceleration of metamorphosis did not accelerate class I expression in this population (data not shown). Erythrocytes began to express class I at day 46 (stages 57–58), and expression

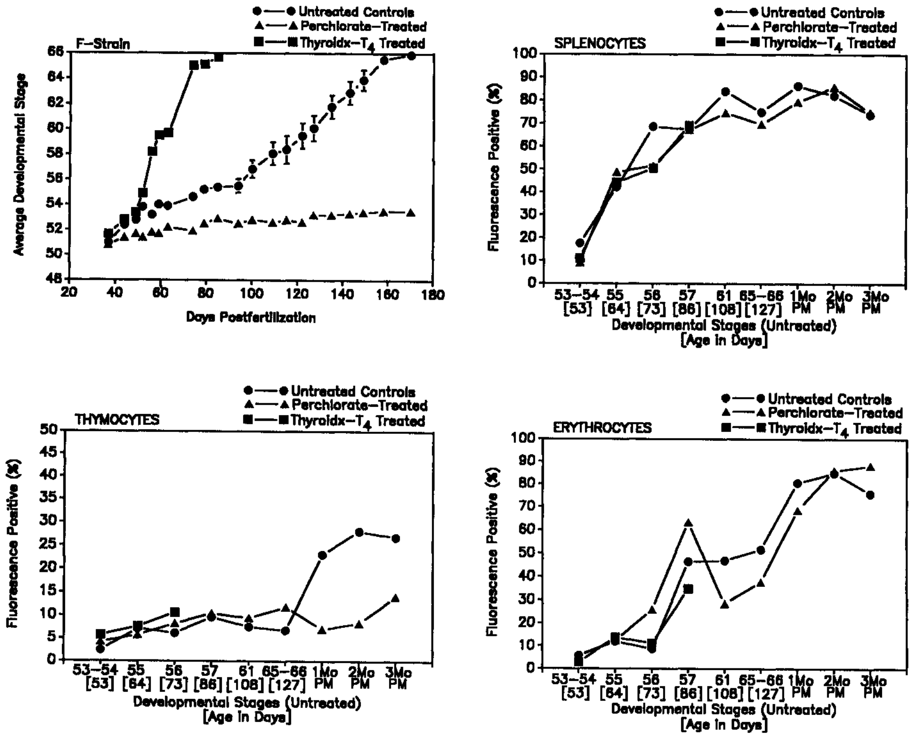

FIGURE 3 Expression of MHC class I antigens determined by flow cytometry in a family of MHC homozygous F-strain animals. Water temperature during development of this family was about 18–20°C, and this family developed more slowly than the progression described by Nieuwkoop and Faber (1967). Upper left shows the developmental stage progression of normal control (closed circles), perchlorate-blocked tadpoles (closed triangles), and thyroidx-$T_4$-treated frogs (closed squares). Upper right, lower left, and lower right show the percent of positively fluorescent cells in spleen, thymus, and blood, respectively. Most data points represent a single pool of cells from 1-5 individuals. The data point for thymocytes at day 86 was omitted because there were too few viable cells for an accurate determination.

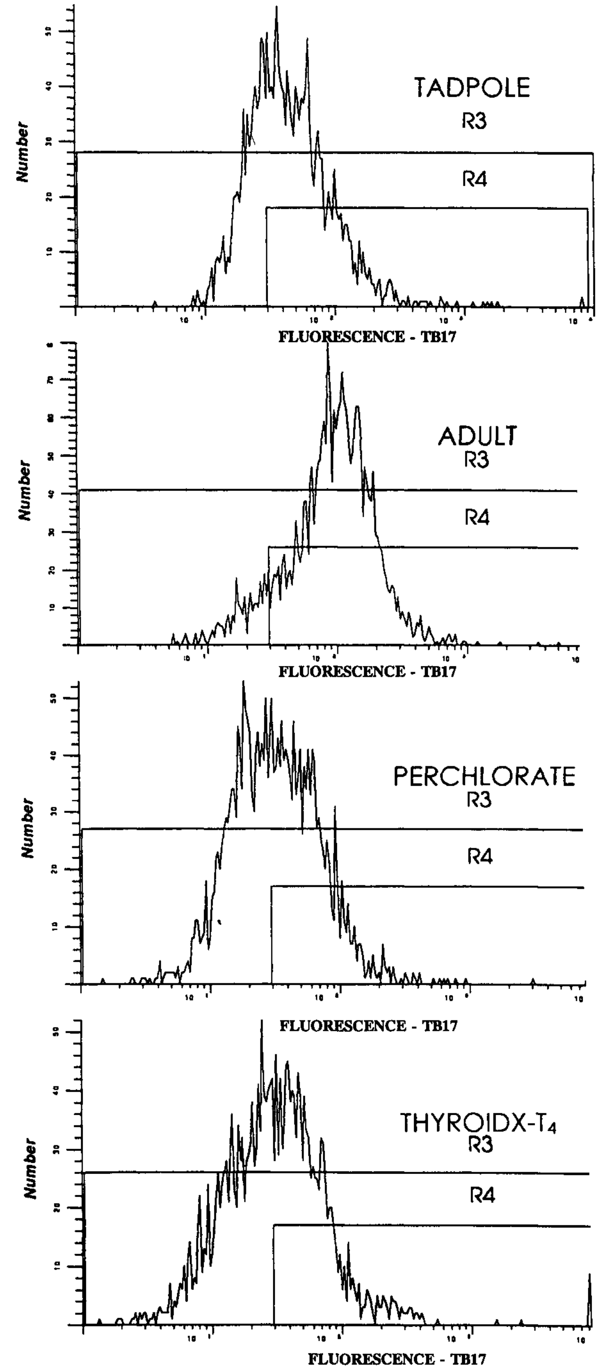

FIGURE 4 Representative fluorescence histograms for splenocytes from normal tadpoles, postmetamorphic adults, perchlorate-blocked tadpoles, and thyroidx-$T_4$-accelerated frogs stained with TB17. Note the shift in fluorescence intensity to the right for adult cells.

increased at later stages. Although $T_4$-treated frogs had already reached stage 62 by day 46, class I expression on erythrocytes was not accelerated (data not shown). Thus, in two families of F-strain frogs examined, class I expression was low or absent until prometamorphic stages 55–56 and increased in all populations to a maximum after metamorphosis. Nevertheless, $T_4$ acceleration did not alter the pattern.

### Relative Fluorescence Intensity of Class I Antigen Expression Detected by Flow Cytometry in the F-Strain

Another advantage of flow cytometry is that one can examine not only the number of cells expressing a specific antigen, but also the relative intensity of expression. Figure 4 and Table I show a comparison of the linear mean fluorescence of splenocytes from normal tadpoles, postmetamorphic adults, perchlorate-treated tadpoles, and thyroidx-$T_4$-treated frogs of the F-strain stained with TB17. Although tadpole splenocytes express class I antigens, mean fluorescence intensity (meanX = the sum of all events at each fluorescence channel divided by the total number of events) is significantly less than that of adults (Student's $t$-test, $p \leq 0.025$). For tadpole splenocytes (stages 53–61, days 53–108), meanX following staining with an irrelevant mAb was 25.1 ± 9.5. When stained with TB17, meanX = 73.0 ± 5.0 (Table I, Fig. 4, top panel).

For adult cells (stages 65–66 to 3 months postmetamorphosis, days 127–224), meanX for control mAb = 16.4 ± 4.6. When stained with TB17, meanX = 103.5 ± 8.4 (Table I). This is reflected in the shift of the fluorescence intensity peak to the right, as shown for adult cells in Fig. 4, second panel from the top. Thus, adult cells are significantly brighter than tadpole cells (Students $t$-test, $p \leq 0.025$).

Splenocytes from perchlorate-treated tadpoles retained the dim staining pattern at all time points examined. MeanX for cells from perchlorate-treated tadpoles stained with the control mAb = 17.8 ± 3.9. MeanX for staining with TB17 = 72.6 ± 5.0 (Table I, Fig. 4, third panel from the top). This is not significantly different from fluorescence intensity expressed by

TABLE I Linear Mean Fluorescence: F-Strain Splenocytes

| Experimental group | Inclusive stages | Age in days | MeanX ± s.e.m. Control mAb | TB17 |
|---|---|---|---|---|
| Tadpoles | 53–54 | 53 | 45.3 | 87.7 |
| Tadpoles | 54–55 | 64 | 51.2 | 80.4 |
| Tadpoles | 56 | 73 | 11.5 | 69.0 |
| Tadpoles | 57 | 86 | 7.9 | 58.7 |
| Tadpoles | 61 | 108 | 9.7 | 69.4 |
| | | | 25.1 ± 9.5 | 73.0 ± 5.0 |
| Adults | 65–66 | 127 | 23.2 | 104.7 |
| Adults | 1 Mo PM | 148 | 10.1 | 101.6 |
| Adults | 2 Mo PM | 182 | 7.2 | 83.2 |
| Adults | 3 Mo PM | 224 | 25.3 | 124.5 |
| | | | 16.4 ± 4.6 | 103.5 ± 8.4 |
| Perchlorate | 52–53 | 53 | 28.0 | 56.6 |
| Perchlorate | 52–53 | 64 | 38.0 | 80.8 |
| Perchlorate | 51–53 | 73 | 32.3 | 97.9 |
| Perchlorate | 53 | 86 | 15.7 | 57.5 |
| Perchlorate | 52 | 108 | 10.1 | 68.4 |
| Perchlorate | 53 | 127 | 9.2 | 57.3 |
| Perchlorate | 53 | 148 | 15.7 | 89.4 |
| Perchlorate | NR | 182 | 8.1 | 66.6 |
| Perchlorate | 55–56 | 224 | 13.2 | 80.0 |
| | | | 17.8 ± 3.9 | 72.6 ± 5.0 |
| Thyroidx-$T_4$ | 58 | 53 | 26.4 | 68.6 |
| Thyroidx-$T_4$ | 61 | 64 | 62.0 | 88.0 |
| Thyroidx-$T_4$ | 64–65 | 73 | 66.4 | 70.9 |
| Thyroidx-$T_4$ | 66 | 86 | 17.6 | 77.4 |
| | | | 43.1 ± 12.3 | 76.2 ± 4.3 |

Linear mean fluorescence (MeanX) for cells stained with each mAb was determined for the spleen-cell population shown in region 3 (R3) of Fig. 4. This includes both fluorescence-negative and fluorescence-positive cells. MeanX for the adult cell population stained with TB17 was significantly greater than that of normal tadpoles, perchlorate-blocked tadpoles, and thyroidx-$T_4$-accelerated frogs (1-tailed Student's *t*-test, $p \leq 0.025$). NR indicates not recorded.

TABLE II Linear Mean Fluorescence: F-Strain Erythrocytes

| Experimental group | Inclusive stages | Age in days | MeanX ± s.e.m. Control mAb | TB17 |
|---|---|---|---|---|
| Tadpoles | 54–55 | 64 | 17.7 | 28.8 |
| Tadpoles | 56 | 73 | 17.9 | 27.4 |
| Tadpoles | 57 | 86 | 13.0 | 20.9 |
| Tadpoles | 61 | 108 | 13.1 | 25.2 |
| Tadpoles | 65–66 | 127 | 10.3 | 28.0 |
| | | | 14.4 ± 1.5 | 25.9 ± 1.4 |
| Adults | 1 Mo PM | 148 | 24.0 | 103.9 |
| Adults | 2 Mo PM | 182 | 12.0 | 64.3 |
| Adults | 3 Mo PM | 224 | 14.5 | 73.9 |
| | | | 16.8 ± 3.7 | 80.7 ± 11.9 |
| Young Perc. | 52–53 | 64 | 18.0 | 30.9 |
| Young Perc. | 51–53 | 73 | 26.0 | 37.9 |
| Young Perc. | 53 | 86 | 8.3 | 26.6 |
| Young Perc. | 52 | 108 | 14.4 | 25.1 |
| Young Perc. | 53 | 127 | 13.1 | 29.7 |
| | | | 16.0 ± 2.9 | 30.0 ± 2.2 |
| Older Perc. | 53 | 148 | 25.5 | 48.8 |
| Older Perc. | NR | 182 | 13.2 | 34.6 |
| Older Perc. | 55–56 | 224 | 22.3 | 49.2 |
| | | | 20.3 ± 3.7 | 44.2 ± 4.8 |
| Thyroidx-$T_4$ | 61 | 64 | 24.0 | 31.5 |
| Thyroidx-$T_4$ | 64–65 | 73 | 23.3 | 39.4 |
| Thyroidx-$T_4$ | 66 | 86 | 13.9 | 27.0 |
| | | | 20.4 ± 3.2 | 32.6 ± 3.6 |

Linear mean fluorescence (MeanX) for cells stained with each mAb was determined for all erythrocytes. This includes both fluorescence-negative and fluorescence-positive cells. MeanX for the adult cells stained with TB17 was significantly greater than that of all other populations stained with TB17. This includes normal tadpoles, younger perchlorate-blocked (Young Perc.) tadpoles, older perchlorate-blocked (Older Perc.) tadpoles, and thyroidx-$T_4$-treated frogs (1-tailed Student's *t*-test, $p \leq 0.05$). MeanX for TB17 stained cells from older perchlorate-blocked frogs (148–224 days of age) was significantly greater than that of normal tadpoles and younger perchlorate-blocked frogs (1-tailed Student's *t*-test, $p \leq 0.025$). NR indicates not recorded.

normal tadpole splenocytes, but significantly less than that of adult splenocytes (Students *t*-test, $p \leq 0.025$).

In this experiment, thyroidx-$T_4$-treated animals metamorphosed at a very early age in comparison with controls. Animals examined at stages 58–66 (days 53–86) might have been expected to express class I antigens in the adult pattern, but they did not. MeanX for control mAb = 43.1 ± 12.3 and the meanX for TB17 was 76.2 ± 4.3 (Table I, Fig. 4, bottom panel). Thus, it appears that metamorphosis was completed before high-level class I expression had developed. It should be noted that some thyroidx-$T_4$-treated frogs from the F X Outbred family were allowed to survive until 1 month postmetamorphosis. At that time, they expressed adult levels of class I (data not shown).

As was observed with splenocytes of untreated control frogs, relative fluorescence intensity increased on erythrocyte populations after metamorphosis (Table II). Increased expression on erythrocytes occurred slightly later than was observed on splenocytes. Although somewhat dimmer than adult splenocytes, adult erythrocytes were about three- to fourfold brighter than tadpole erythrocytes (Table II). Erythrocytes from younger perchlorate-treated tadpoles (days 53–127) were approximately of the same fluorescence intensity as normal untreated tadpoles (Table II). However, older perchlo-

TABLE III Linear Mean Fluorescence F-Strain Thymocytes

| Experimental group | Inclusive stages | Age in days | MeanX ± s.e.m. | |
|---|---|---|---|---|
| | | | Control mAb | TB17 |
| Tadpoles | 53–54 | 53 | 8.6 | 11.8 |
| Tadpoles | 54–55 | 64 | 8.1 | 14.1 |
| Tadpoles | 56 | 73 | 6.4 | 10.1 |
| Tadpoles | 57 | 86 | 5.2 | 12.1 |
| Tadpoles | 61 | 108 | 6.9 | 12.8 |
| Tadpoles | 65–66 | 127 | 5.1 | 16.1 |
| | | | 6.7 ± 0.6 | 12.8 ± 0.8 |
| Adults | 1 Mo PM | 148 | 11.8 | 28.1 |
| Adults | 2 Mo PM | 182 | 12.0 | 21.4 |
| Adults | 3 Mo PM | 224 | 18.8 | 29.9 |
| | | | 14.2 ± 2.3 | 26.5 ± 2.6 |
| Perchlorate | 52–53 | 53 | 6.8 | 18.5 |
| Perchlorate | 52–53 | 64 | 8.1 | 11.9 |
| Perchlorate | 51–53 | 73 | 5.7 | 13.1 |
| Perchlorate | 53 | 86 | 6.8 | 10.1 |
| Perchlorate | 52 | 108 | 7.4 | 14.3 |
| Perchlorate | 53 | 127 | 4.2 | 9.8 |
| Perchlorate | 53 | 148 | 10.0 | 14.2 |
| Perchlorate | NR | 182 | 11.8 | 15.0 |
| Perchlorate | 55–56 | 224 | 10.9 | 16.5 |
| | | | 8.0 ± 0.8 | 13.7 ± 0.9 |
| Thyroidx-$T_4$ | 58 | 53 | 5.4 | 12.6 |
| Thyroidx-$T_4$ | 61 | 64 | 8.1 | 20.5 |
| Thyroidx-$T_4$ | 64–65 | 73 | 17.1 | 31.9 |
| | | | 10.2 ± 3.5 | 21.7 ± 5.6 |

Linear mean fluorescence (MeanX) for cells stained with each mAb was determined for all thymocytes. This includes both fluorescence-negative and fluorescence-positive cells. MeanX for the adult cell population stained with TB17 was significantly greater than that of normal tadpoles, and perchlorate-blocked tadpoles (1-tailed Student's *t*-test, $p \leq 0.0005$). NR indicates not recorded.

rate-treated tadpoles showed an apparent increase in relative fluorescence intensity (Table II). As was observed with splenocytes, the meanX of erythrocytes of Thyroidx-$T_4$-treated frogs did not show increased intensity of staining with TB17 monoclonal at the time points examined (up to 86 days of age) (Table II).

Thymocyte populations were very dimly positive at larval stages and fluorescence intensity increased slightly in postmetamorphic adults (average linear mean fluorescence = 12.8 ± 0.8 for larval cells and 26.5 ± 2.6 for the adult population; Table III). There was no evidence for increased expression on thymocytes of older perchlorate-treated frogs (Table III). The increased expression on postmetamorphic thymocytes reflects an increase in the number of positive cells rather than increased expression on individual cells (data not shown).

### DISCUSSION

These studies examined the possible role of thyroid hormones in the regulation of expression of MHC class I antigens. In the families of frogs examined, class I was first detected in the spleen of untreated control frogs at stage 55 when animals were reared at 22–24°C and developed according to the Nieuwkoop and Faber schedule of stages (Nieuwkoop and Faber, 1967). When animals were reared at slightly lower temperatures and developed more slowly, class I could be detected as early as stages 53–54. Neither inhibition of metamorphosis nor acceleration of metamorphosis altered the time of onset of class I expression (Figs. 1 and 3).

On splenocytes and erythrocytes of untreated control frogs of the F-strain, relative fluorescence intensity, expressed as linear mean fluorescence, was increased at postmetamorphic stages. Because the percentage of positive cells in the spleen remained about the same at late larval stages and after metamorphosis, increased fluorescence reflects increased expression on individual cells. Because the splenocytes and erythrocytes recovered from older perchlorate-blocked frogs (148–224 days of age) did not show the increased expression of class I antigens characteristic of postmetamorphic adults, thyroid hormones may be involved in regulation of the level of class I expression. However, in young thyroidx-$T_4$-accelerated frogs, splenocytes and erythrocytes also retained the larval pattern of dim fluorescence intensity. Thus, although a significant number of splenocytes and erythrocytes were class I positive, the switch to higher-intensity expression may depend on other metamorphosis-associated changes in addition to thyroid hormones. Splenocytes and erythrocytes from thyroidx-$T_4$-accelerated frogs that were allowed to survive for 1 month postmetamorphosis showed adult levels of class I expression (data not shown). Therefore, following thyroid-hormone-driven metamorphosis, class I expression can be upregulated in these animals, but it was not increased in perchlorate-inhibited permanent tadpoles. On thymocytes, class I expression was dim even in postmetamorphic animals, but an increased number of thymocytes became positive after metamorphosis.

In older perchlorate-treated permanent tadpoles, erythrocytes showed increased intensity of class I expression, but expression did not equal adult levels. This result was observed previously by Flajnik and Du Pasquier (1988).

A previous study of the skin allograft rejection capabilities of thyroidx and $T_4$-accelerated frogs showed that the accelerated frogs had impaired abilities to reject grafts differing by one MHC haplotype or minor histoincompatibilities (Rollins-Smith et al., 1988). We hypothesized that these results were due to emergence of a wave of thymocytes in thyroidx-$T_4$-accelerated frogs out of synchrony with expression of class I MHC antigens. Supporting this idea, we have shown in the present study that class I is not expressed at high levels on any of the cell populations examined until after metamorphosis, and frogs forced to undergo premature metamorphosis were unable to upregulate class I expression until sometime after metamorphosis. This would suggest that effective CD8-mediated T-cell-dependent cytotoxicity probably cannot develop until after metamorphosis in frogs. Cytotoxicity at earlier stages may depend on natural killer (NK)–type cells. Perhaps low-level expression of class I antigens at larval stages may be necessary to avoid destruction by NK-like cells (reviewed in Ljunggren and Karre, 1990). Nothing is known about the ontogeny of NK-like cells in *Xenopus*.

Because onset of expression was not accelerated in $T_4$-treated frogs or delayed in metamorphosis-inhibited frogs, it is unlikely that thyroid hormones are the developmental cues for onset of expression of class I, although they may be involved in the upregulation of class I expression that occurs at metamorphosis. Differences in the pattern of expression in different subpopulations of cells suggest a complex pattern of regulation of expression of class I antigens during ontogeny.

Other signals that regulate class I expression during ontogeny in *Xenopus* remain a scientific puzzle. Although the potential regulatory region upstream of the coding region of the class I gene has been sequenced (Flajnik, unpublished), there are no obvious motifs that would reveal the nature of the regulatory signals. It is also not clear why class I antigens are expressed weakly in late larval life and expression upregulated 1–3 months postmetamorphosis. Flajnik and Du Pasquier (1988) showed that a high level of class I expression was found in the newly emerging adult erythrocyte population after metamorphosis. Persisting larval erythrocytes expressing lower levels of class I antigen disappeared soon after metamorphosis. Increased expression on splenic lymphocytes may reflect the development of a new adult lymphocyte population as well. Lymphocyte populations are expanding rapidly after metamorphosis (Du Pasquier and Weiss, 1973; Rollins-Smith et al., 1984). Class I expression is central to the ability of the vertebrate immune system to recognize and remove virus-infected cells, and understanding what regulates class I expression remains an important research goal. *Xenopus* offers a unique model system to investigate regulation of class I expression, especially during ontogeny.

### MATERIALS AND METHODS

### Frogs

MHC homozygous F-strain frogs expressing class I MHC antigens recognized by the monoclonal antibody designated TB17 (Flajnik et al., 1991b) or outbred frogs (*Xenopus I*, Ann Arbor, MI) selected for expression of the TB17 determinant were used in these studies. Adult breeding pairs were induced to breed by injection of human chorionic gonadotropin (Rollins-Smith and Blair, 1990b). Offspring were reared at a density of 8–10 tadpoles per 4 l of dechlorinated tap water at 18–22°C unless otherwise indicated in figure legends. The water was changed three times weekly, and tadpoles were fed finely powdered nettle leaf. After metamorphosis, young frogs were fed freeze-dried Tubifex worms and small particles of commercial trout chow (Purina, St. Louis). Adult frogs were fed ground beef heart and commercial trout chow.

### Thyroidectomy and Thyroxine Treatment to Accelerate Metamorphosis

Thyroidectomies were generally performed at stages 50–51 (days 15–25) according to the method of Nagata (1976) with slight modifications, as previously described (Rollins-Smith et al., 1988). To accelerate metamorphosis, thyroidx tadpoles were allowed to develop until they were about 28 days of age and then treated with a regimen of increasing concentrations of $T_4$ in the water, as previously described (Rollins-Smith et al., 1988). Briefly, they received 0.3 $\mu$g/l $T_4$ from days 28–35; 1.0 $\mu$g/l $T_4$ from days 35–42. If the animals had reached stage 54, they received 20 $\mu$g/l $T_4$ from days 42–56. (If the animals had not reached stage 54, they were treated for an additional week with 1 $\mu$g/l $T_4$.) This relatively high concentration of $T_4$ advanced them through a precocious metamorphosis. After day 56, they were treated continuously with 5 $\mu$g/l $T_4$.

### Sodium Perchlorate Treatment to Inhibit Metamorphosis

Beginning at days 22–25, some larvae were placed in dechlorinated tap water containing 1 g/l sodium perchlorate (Sigma, St. Louis). Water containing perchlorate was changed and the animals were fed three times weekly.

### Preparation of Cells for Staining and Analysis

When experimental animals reached a specific developmental stage, they were sacrificed, and cells from blood, thymus, and spleen were collected, as previously described (Rollins-Smith and Blair, 1990b). Blood was collected from the exposed heart. Spleen and thymuses were dissociated with fine forceps in about 300 $\mu$l of Leibovitz (L-15) medium with supplements, as previously described (Rollins-Smith and Blair, 1990b). For counting, cells were washed once and resuspended in a known volume. An aliquot was removed, stained with trypan blue, and viable cells counted with a hemocytometer counting chamber.

### Anti-Class I Monoclonal Antibody

The monoclonal antibody (mAb), TB17, specific for the MHC class I determinant expressed by F-strain animals has been previously described (Flajnik et al., 1991b). A supernatant from the hybridoma cell line containing approximately 100 $\mu$g/ml of antibody was preserved with 0.1% sodium azide and used for staining without further dilution.

### Indirect Microscopy

Approximately $5 \times 10^5$ thymocytes, splenocytes, or erythrocytes were stained with 20 $\mu$l of TB17 for 20 min at 4°C. Cells were washed with 500 $\mu$l of amphibian phosphate buffered saline (APBS) (6.6 g NaCl, 1.15 g Na2HPO4, and 0.2 g KH2PO4 in 1000 ml of glass distilled water, pH adjusted to 7.5) containing 2% fetal calf serum and 0.2% sodium azide (PFA). The cells were counterstained with FITC-conjugated goat anti-mouse immunoglobulin (FITC-Gt-anti-mouse Ig) antisera (Southern Biotechnology Associates, Birmingham, AL) at 100 $\mu$g/ml in PFA for an additional 20 min at 4°C. After the second antibody was washed out with PFA, the cells were resuspended at $1 \times 10^6$/ml in PFA and $1 \times 10^5$ were centrifuged onto microscopic slides with a cytocentrifuge (Shandon Southern Instrument, Pittsburgh). After air drying, the adherent cells were fixed for 20 min in a very cold (-20°C) 95% ethanol 5% glacial acetic acid solution. After rehydration in APBS and mounting, the slides were examined at 500–800 × magnification with a Leitz Orthoplan fluorescence microscope with epillumination. The percentage of cells with positive immunofluorescence was determined after counting approximately 100–500 cells of lymphocyte morphology.

### Flow Cytometry

Cells were prepared and stained as described earlier. In addition, a second aliquot of cells was stained with an isotype-matched control mAb ($IgG_1$, k) followed by FITC Gt-anti-mouse Ig. The isotype control antibody is directed at a determinant of *Xenopus* vitellogenin and does not react with cell-surface antigens. It was a kind gift of Dr. Akira Kawahara. After staining, cells were fixed for at least 1 hr in PFA containing 1% formalin. Some of the cells were analyzed on a Coulter EPICS 752 flow cytometer. Before completion of the project, a Becton-Dickinson FACSTAR Plus and a BD FACScan were purchased. The BD machines replaced the EPICS machine, and remaining samples were analyzed on one of these machines. Viability was determined by exclusion of 7-aminoactinomycin D (7-AAD) (Sigma) from cell populations. 7-AAD was added prior to fixation. Electronic gates were defined on viability, forward, and 90°C light-scatter parameters so as to select 5,000–20,000 viable cells for analysis. Data stored in a list mode was analyzed using the Win-List program (Verity Software, Topsham, ME). Linear mean fluorescence (MeanX), defined as the sum of all events at each fluorescence channel divided by the total number of events, was computed for class I (TB17) stained cells or cells stained with the isotype control antibody.

### Acknowledgments

This research was supported by grants DCB-8710235, DCB-9004666, and MCB-9421349 from the National Science Foundation. We acknowledge Dr. Wayne Green and Dr. Jim Price and the flow cytometry laboratory of the Veteran's Administration Medical Center of Nashville for assistance with flow cytometry. We thank Ruth Moore for assistance in preparation of the manuscript.

### References

Du Pasquier L., and Flajnik M.F. (1990). Expression of MHC class II antigens during *Xenopus* development. Dev. Immunol. **1**: 85–95.

Du Pasquier L., Schwager J., and Flajnik M.F. (1989). The immune system of *Xenopus*. Ann. Rev. Immunol. **7**: 251–275.

Du Pasquier L., and Weiss N. (1973). The thymus during the ontogeny of the toad *Xenopus laevis*: Growth, membrane-bound immunoglobulins and mixed lymphocyte reaction. Eur J. Immunol. **3**: 773–777.

Flajnik M.F., Canel C., Kramer J., and Kasahara M. (1991a). Evolution of the major histocompatibility complex: Molecular cloning of major histocompatibility complex class I from the amphibian *Xenopus*. Proc. Natl. Acad. Sci. USA **88**: 537–541.

Flajnik M.F., and Du Pasquier L. (1988). MHC class I antigens as surface markers of adult erythrocytes during the metamorphosis of *Xenopus*. Dev. Biol. **128**: 198–206.

Flajnik M.F., and Du Pasquier L. (1990). The major histocompatibility complex of frogs. Immunol. Rev. **113**: 47–63.

Flajnik M.F., Du Pasquier L., and Cohen N. (1985). Immune responses of thymus/lymphocyte embryonic chimeras: Studies on tolerance and major histocompatibility complex restriction in *Xenopus*. Eur. J. Immunol. **15**: 540–547.

Flajnik M.F., Ferrone S., Cohen N., and Du Pasquier L. (1990). Evolution of the MHC: Antigenicity and unusual tissue distribution of *Xenopus* (frog) class II molecules. Mol. Immunol. **27**: 451–462.

Flajnik M.F., Hsu E., Kaufman J.F., and Du Pasquier L. (1987). Changes in the immune system during metamorphosis of *Xenopus*. Immunol. Today **8**: 58–64.

Flajnik M.F., Kaufman J.F., Hsu E., Manes M., Parisot R., and Du Pasquier L. (1986). Major histocompatibility complex encoded class I molecules are absent in immunologically competent *Xenopus* before metamorphosis. J. Immunol. **137**: 3891–3899.

Flajnik M.F., Kaufman J.F., Riegert P., and Du Pasquier L. (1984). Identification of class I major histocompatibility complex encoded molecules in the amphibian *Xenopus*. Immunogenetics **20**: 433–442.

Flajnik M.F., Taylor E., Canel C., Grossberger D., and Du Pasquier L. (1991b). Reagents specific for MHC class I antigens of *Xenopus*. Amer. Zool. **31**: 580–591.

Horton J.D. (1994). Amphibians. In Immunology: A comparative approach, Turner R.J., Ed. (New York: John Wiley), pp. 101–136.

Kaufman J.F., Flajnik M.F., and Du Pasquier L. (1985b). *Xenopus* MHC class II molecules. II. Polymorphism as determined by two-dimensional gel electrophoresis. J. Immunol. **134**: 3258–3264.

Kaufman J.F., Flajnik M.F., Du Pasquier L., and Riegert P. (1985a). *Xenopus* MHC class II molecules. I. Identification and structural characterization. J. Immunol. **134**: 3248–3257.

Leloup, J. and Buscaglia M. (1977). La triiodothyronine, hormone de la métamorphose des Amphibians. CR. Acad. Sci. Paris **284D**: 2261–2263.

Ljunggren H.G., and Karre K. (1990). In search of the "missing self": MHC molecules and NK cell recognition. Immunol. Today **11**: 237–244.

Nagata S. (1976). Immune response against skin allograft and rabbit red blood cells in the metamorphosing and metamorphosis-inhibited *Xenopus laevis*. J. Fac. Sci. Hokkaido Univ. **20**: 183–191.

Nieuwkoop P.D., and Faber J. (1967). Normal Table of *Xenopus laevis* Daudin (Amsterdam: North-Holland).

Rollins-Smith L.A., and Blair P. (1990a). Expression of class II major histocompatibility complex antigens on adult T cells in *Xenopus* is metamorphosis-dependent. Dev. Immunol. **1**: 97–104.

Rollins-Smith L.A., and Blair P. (1990b). Contribution of ventral blood island mesoderm to hematopoiesis in postmetamorphic and metamorphosis-inhibited *Xenopus laevis*. Dev. Biol. **142**: 178–183.

Rollins-Smith L.A., Blair P.J., and Davis A.T. (1992). Thymus ontogeny in frogs: T-cell renewal at metamorphosis. Dev. Immunol. **2**: 207–213.

Rollins-Smith L.A., and Cohen N. (1995). Metamorphosis: An immunologically unique period in the life of the frog. In Metamorphosis: Postembryonic reprogramming of gene expression in amphibian and insect cells, Gilbert L.I., Atkinson B.G., and Tata J., Eds. (New York: Academic Press), pp. 625-646.

Rollins-Smith L.A., Parson S.C.V., and Cohen N. (1984). During frog ontogeny, PHA and Con A responsiveness of splenocytes precedes that of thymocytes. Immunology **52**: 491–500.

Rollins-Smith L.A., Parsons S.C.V., and Cohen N. (1988). Effects of thyroxine-driven precocious metamorphosis on maturation of adult-type allograft rejection responses in early thyroidectomized frogs. Differentiation **37**: 180–185.

Sato K., Flajnik M.F., Du Pasquier L., Katagiri M., and Kasahara M. (1993). Evolution of the MHC: Isolation of class II $\beta$-chain cDNA clones from the amphibian *Xenopus laevis*. J. Immunol. **150**: 2831–2843.

Schwartz R.H. (1984). The role of gene products of the major histocompatibility complex in T cell activation and cellular interactions. In Fundamental immunology, Paul W.E., Ed. (New York: Raven Press), pp. 379–438.

Shum B.P., Avila D., Du Pasquier L., Kasahara M., and Flajnik M.F. (1993). Isolation of a classical MHC class I cDNA from an amphibian. Evidence for only one class I locus in the *Xenopus* MHC. J. Immunol. **151**: 5376–5386.

Turpen J.B., and Smith P.B. (1989). Precursor immigration and thymocyte succession during larval development and metamorphosis in *Xenopus*. J. Immunol. **142**: 41–47.

